# Hemobilia due to Hepatic artery pseudoaneurysm secondary to collateral circulation formation after liver trauma: a case report

**DOI:** 10.1186/s12893-021-01078-6

**Published:** 2021-02-02

**Authors:** Qiqi Wu, Qianling Sun, Bin Mei

**Affiliations:** Department of Hepatic Surgery, Tongji Hospital, Tongji Medical College, Huazhong University of Science and Technology, Wuhan, 430030 China

**Keywords:** Liver trauma, Hepatic artery ligation, Collateral circulation, Hemobilia, Pseudoaneurysm

## Abstract

**Background:**

Hemobilia due to rupture of hepatic artery pseudoaneurysm and recurrent hemorrhage caused by hepatic artery collateral circulation are both rare complications after liver trauma. There have been a number of separate reports of both complications, but no cases have been reported in which the two events occurred in the same patient. Here we report a recurrent hemorrhage in the bile duct due to hepatic artery pseudoaneurysm secondary to collateral circulation formation after hepatic artery ligation in a patient with liver trauma.

**Case presentation:**

A 52-year-old male patient was admitted to our hospital for liver trauma (Grade IV according to the American Association for the Surgery of Trauma (AAST) grading system) with active bleeding after a traffic accident. Hepatic artery ligation was performed for hemostasis. Three months after the surgery, the patient was readmitted for melena and subsequent hematemesis. Selective angiography examination revealed the formation of collateral circulation between the superior mesenteric artery and right hepatic artery. Moreover, a ruptured hepatic artery pseudoaneurysm was observed and transcatheter arterial embolization (TAE) was performed for hemostasis at the same time. After the treatment, the patient recovered very well and had an uneventful prognosis until the last follow-up.

**Conclusion:**

For patients with hepatic trauma, the selection of the site of hepatic artery ligation and the diagnosis and treatment methods of postoperative biliary hemorrhage are crucial for the prognosis of the disease.

## Background

The liver is the largest substantial organ in the human body, and the incidence of liver rupture ranks third among abdominal injuries, accounting for approximately 15–20% [1]. The anatomical features of the liver determine that liver trauma is associated with damage to large blood vessels and other organs; thus, liver rupture has a high mortality. It has been reported that the mortality due to severe liver rupture can reach 30–50% [2].

Hepatic artery pseudoaneurysm (HPA) with biliary hemorrhage is a very rare but critical complication after liver trauma, with an incidence of approximately 1.2–4% [3, 4]. If HPA is not diagnosed or treated in time, the prognosis can be very poor or even fatal. The first case [5] of pseudoaneurysm of the hepatic artery was reported in 1819, and dozens of cases related to HPA have since been reported. Due to its extremely low incidence, most of the relevant studies are case reports, and clinical studies with large samples remain lacking. The mechanism of HPA is related to hepatic artery and bile duct injury, and local inflammation [4] also plays a very important role in its formation. The most common clinical manifestations of HPA with hemobilia are right upper abdominal pain, jaundice and gastrointestinal bleeding [6]. Usually, there is an approximately four-week interval between the appearance of biliary hemorrhage and the liver trauma or iatrogenic surgery that causes hemobilia [7, 8]. Therefore, it can be difficult to make an accurate diagnose.

There have been a number of isolated cases of recurrent hemorrhage caused by hepatic artery collateral circulation or rupture of pseudoaneurysm after liver trauma. However, no cases have been reported in which the two events occurred in the same patient and eventually ended in hemobilia.

## Case presentation

**Fig. 1:**
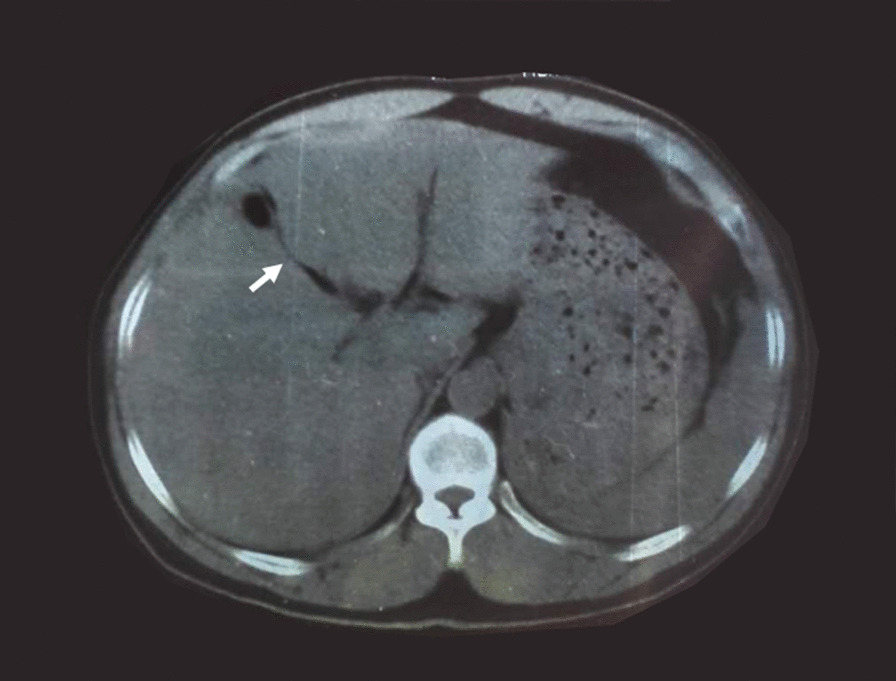
Abdominal computed tomography image showing liver lacerations (arrow)

**Fig. 2:**
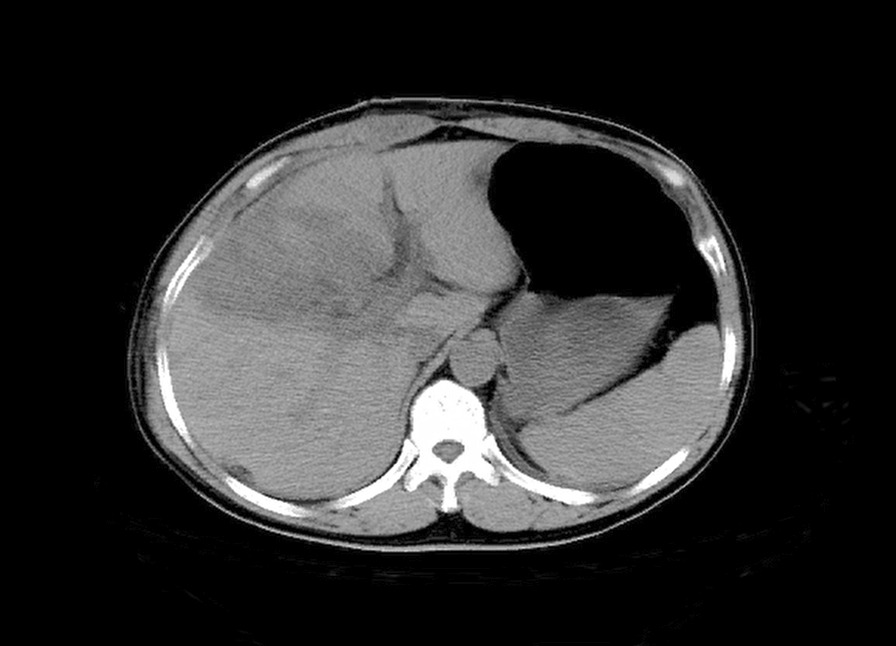
Postoperative abdominal CT scan

**Fig. 3:**
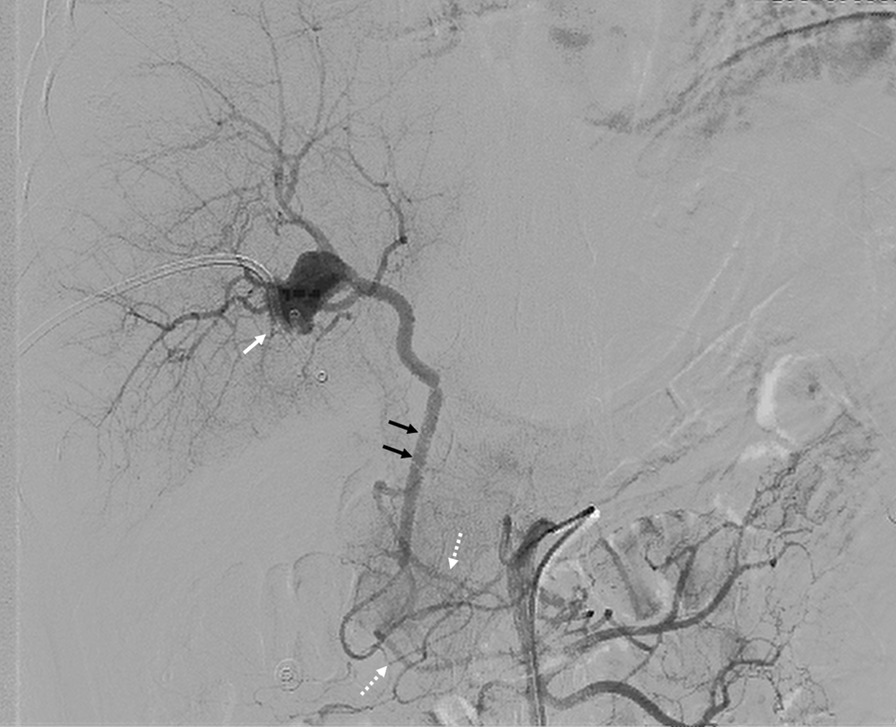
Superior mesenteric angiogram demonstrating collateral circulation and hepatic artery pseudoaneurysm. Note anterior/posterior pancreaticoduodenal arch (white dotted arrows), gastroduodenal artery (black solid arrows), and pseudoaneurysm (white solid arrows)

**Fig. 4:**
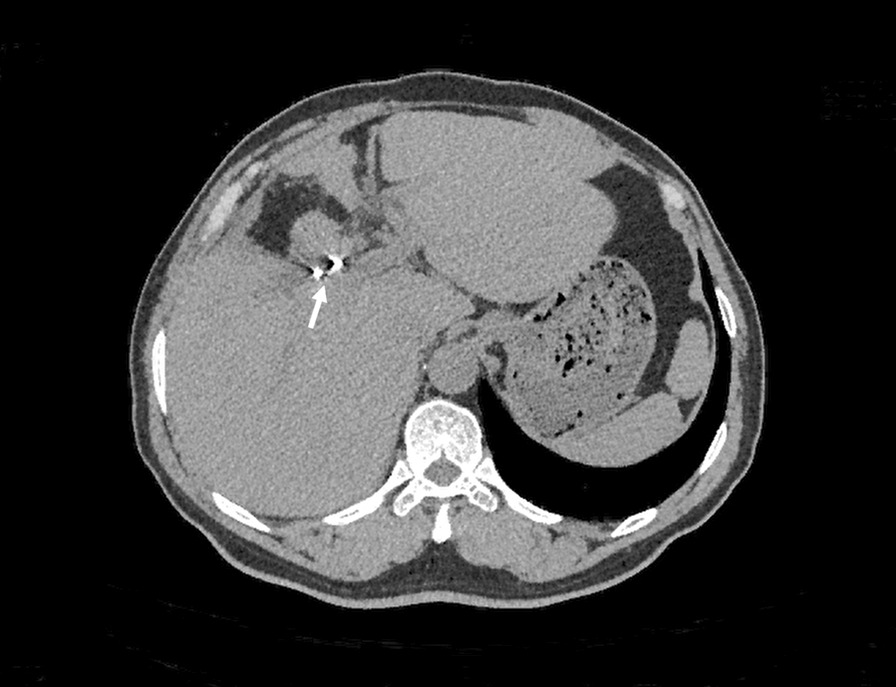
Abdominal CT scan of the last follow-up. Note the spring coil of the pseudoaneurysm

A 52 year-old man was admitted to our hospital 5 h after exploratory laparotomy for abdominal trauma. The patient’s abdomen was injured in a car accident more than 10 h earlier. He was first treated in a local hospital, abdominal computed tomography indicated liver rupture and peritoneal effusion (Fig. 1). Emergency laparotomy was performed for hemostasis. At laparotomy, a Grade IV liver injury was seen with multiple lacerations of both the right and left liver lobes. After suturing the liver lacerations and removing the gallbladder, massive hemorrhage occurred in the gallbladder fossa and the first hepatic hilum. Gauze packing was performed for emergency hemostasis. However, the patient still had signs of active bleeding after surgery: (i) hypotension: systolic blood pressure fluctuated between 60 and 87 mm Hg; (ii) blood drainage fluid: 1000 ml of bloody fluid was drained out of the drainage tube of Wen’s hole in 3 h. Therefore, he was referred to our hospital for further examination and treatment. The patient had no previous underlying disease. Physical examination revealed hemorrhagic shock: the patient was in a coma, with endotracheal intubation mechanical ventilation to maintain respiration; pulse: 112 beats per minute, blood pressure: 71/42 mm Hg. A surgical incision of approximately 25 cm and three drainage tubes were visible in the right rectus abdominis. Routine blood test showed that the hemoglobin level was 58 (130–175) g/L. After anti-shock therapy, emergency surgery was performed, due to the large amount of blood loss and difficulty in dissociating the first hepatic hilum, only common hepatic artery ligation and liver repair were performed. A postoperative CT scan revealed no obvious perihepatic effusion or celiac vascular abnormalities (Fig. 2). Some complications such as postoperative pulmonary infection, transient liver insufficiency, biliary leakage and peritoneal effusion occurred in the patient. After providing respiratory support, fluid replenishment, anti-infection and some other treatments, the patient recovered very well and was discharged home on the 20th postoperative day with a drainage tube in Wen's hole from which 100 ml of dark green liquid was drained every day. Approximately 3 months after the operation, the patient was readmitted to our hospital for abdominal distension accompanied by melena for two days. On the third day after admission, the patient had a sudden hematemesis of about 200 ml without obvious inducement, followed by 200 ml of melena and 800 ml of bloody fluid drained from the drainage tube of Wen's hole. Further selective angiography examination (Fig. 3) revealed the establishment of collateral circulation of the superior mesenteric artery—lower pancreaticoduodenal artery—anterior/posterior pancreaticoduodenal arch—superior pancreaticoduodenal artery—gastroduodenal artery—right hepatic artery. Moreover, a ruptured hepatic artery pseudoaneurysm was observed and transcatheter arterial embolization (TAE) was performed for hemostasis at the same time. The patients recovered very well after TAE treatment and no harmful events occurred during the follow-up period. The last follow-up (Fig. 4) was performed more than four years after the operation.

## Discussion and conclusions

Recurrent hemorrhage caused by hepatic artery collateral circulation or rupture of pseudoaneurysm is a rare complication after liver trauma [9, 10] There are a number of separate reports of both complications, but what is interesting in this case is that the two complications occurred in the same patient and there could be a causal relationship between them.

Studies have shown that there are extensive extrahepatic blood supplies to the liver. Michels [11] described 26 possible collateral pathways in a surgical anatomy study based on 200 cadavers: (i) 10 vagal or accessory hepatic arteries from the superior mesenteric artery, left gastric artery or other origin; (ii) 6 extrahepatic channels connected with the hepatic artery, including the gastroepiploic artery and the right gastric artery; and (iii) 10 external abdominal arteries supplying blood, such as the subdiaphragmatic artery and internal mammary artery. After the hepatic artery is ligated, these collateral vessels can provide adequate blood supply to the liver, but the regularity of the opening of these collateral vessels is not clear. In the case described above, only common hepatic artery ligation was performed for hemostasis. However, the right hepatic artery was reopened due to the collateral circulation of the superior mesenteric artery—inferior pancreatoduodenal artery—anterior/posterior arcuate—superior pancreatoduodenal artery—gastroduodenal artery—right hepatic artery, resulting in rebleeding of the right hepatic artery. Moreover, combined with long-term local biliary leakage, pseudoaneurysm of the right hepatic artery formed gradually by wrapping the surrounding bile duct and other tissues. Therefore, for patients with hemorrhagic liver injury requiring hepatic artery ligation, we recommend that intraoperative angiography be performed to determine the best site for ligation. If the above medical conditions can’t be met, we recommend that the gastroduodenal artery should be ligated at the same time when performing ligation of the common hepatic artery or the proper hepatic artery to reduce the opportunity for rebleeding caused by collateral circulation of the gastroduodenal artery [12–14]. Similar to the results in this paper, some authors [2,^ 13^–15] have proposed that combined ligation such as combined ligation of the proper hepatic artery, the common hepatic artery and the gastroduodenal artery has better hemostatic effects than single common hepatic artery or proper hepatic artery ligation. They emphasize that, if the gastroduodenal artery is not ligated, the proper hepatic artery and the right and/or left hepatic artery must be ligated together to achieve hemostasis. However, high quality studies with large samples remain lacking for verification.

Rupture of hepatic artery pseudoaneurysm is a very rare cause of biliary hemorrhage. The most common causes of HPA are liver trauma and iatrogenic injury (such as laparoscopic cholecystectomy, liver biopsy, liver transplantation and biliary puncture). Due to changes in the current medical environment, iatrogenic injury has replaced hepatic trauma as the main cause of pseudoaneurysm of the hepatic artery [16, 17]. Most cases of HPA reported before usually occurred after liver trauma and were treated by TAE or liver artery ligation. However, the case reported in our article occured three months after liver artery ligation, greatly increasing the difficulty in diagnosis and treatment. The exact mechanisms of HPA remain unclear; however, direct hepatic artery and bile duct injury during surgery or hepatic trauma could play a role in the formation of HPA. Some authors have insisted that inflammatory reactions, such as bile leakage, also play an important role in the creation of HPA. Blood leaks from the ruptured hepatic artery, combined with local inflammatory reactions, such as bile leakage, the bile duct and other tissues are gradually wrapped to form a cavity. Continuous bleeding of the hepatic artery and inflammatory reaction gradually increase the pressure of the cavity, eventually leading to rupture into the biliary tract or abdominal cavity [3, 18]. Usually, there is a four-week interval between the appearance of biliary hemorrhage and the liver trauma or iatrogenic surgery that causes it, so sometimes it is very difficult to identify where the bleeding comes from. Quincke’s triad (gastrointestinal bleeding, right upper abdominal pain, jaundice) is the most common clinical symptom. Endoscopy and contrast enhanced CT can be used for diagnosing hemobilia and the formation of the pseudoaneurysms; however, the site of bleeding cannot be accurately located by these modalities. Selective arteriography remains the most accurate examination for the diagnosis of hemobilia and the underlying cause of hemorrhage, with sensitivity up to 90% [17–19]. Moreover, TAE is now the first line treatment for hemostasis due to its high hemostatic rate (approximately 80%), good tolerance and low risk [14,^ 20^, 21]. When TAE treatment fails, surgical treatments such as hepatic artery ligation and hepatectomy should be considered [22–24].

In conclusion, the establishment of collateral circulation after hepatic artery ligation accompanied by hemobilia due to hepatic artery pseudoaneurysm in patients with liver trauma is very rare in clinical practice. Gastroduodenal artery ligation should be considered when performing hepatic artery ligation to reduce the opportunity for rebleeding caused by collateral circulation. Selective angiography and TAE should be the first-line choices for the treatment of patients with suspected biliary hemorrhage caused by rupture of hepatic artery pseudoaneurysm. This case is helpful in deepening the understanding of liver diseases, and it has important clinical significance for guiding the diagnosis and treatment of related liver diseases.

## Data Availability

The data and materials, including all the radical pictures before and after surgery for the patient are included within the article.

## Abbreviations

AAST: American Association for Surgery of Trauma
TAE: Transcatheter Arterial Embolization
HPA: Hepatic Artery Pseudoaneurysm
CT: Computed Tomography
